# GeneXpert HIV-1 quant assay, a new tool for scale up of viral load monitoring in the success of ART programme in India

**DOI:** 10.1186/s12879-017-2604-5

**Published:** 2017-07-21

**Authors:** Smita Kulkarni, Sushama Jadhav, Priyanka Khopkar, Suvarna Sane, Rajkumar Londhe, Vaishali Chimanpure, Veronica Dhilpe, Manisha Ghate, Rajendra Yelagate, Narayan Panchal, Girish Rahane, Dilip Kadam, Nitin Gaikwad, Bharat Rewari, Raman Gangakhedkar

**Affiliations:** 1 Department of Virology, National AIDS Research Institute, Plot No 73, G-block, M I D C, Bhosari, Pune, Maharashtra 411026 India; 2 Byramjee Jeejeebhoy Medical College (BJMC), Sassoon General Hospital, Jai Prakash Narayan Road, Near Pune Railway Station, Pune, Maharashtra 411001 India; 3 Yashwantrao Chavan Memorial Hospital (YCM), No.476/2692, Sant Tukaram Nagar, Pimpri, Pimpri-Chinchwad, Pune, Maharashtra 411018 India; 4 World Health Organization (WHO) Country office for India, World Health House, Indraprastha Estate, Mahatma Gandhi Marg, New Delhi, Delhi 110002 India

**Keywords:** GeneXpert HIV-1 quant, Abbott RealTime PCR, HIV-1, Viral load, Point of care technologies, ART

## Abstract

**Background:**

Recent WHO guidelines identify virologic monitoring for diagnosing and confirming ART failure. In view of this, validation and scale up of point of care viral load technologies is essential in resource limited settings.

**Methods:**

A systematic validation of the GeneXpert® HIV-1 Quant assay (a point-of-care technology) in view of scaling up HIV-1 viral load in India to monitor the success of national ART programme was carried out. Two hundred nineteen plasma specimens falling in nine viral load ranges (<40 to >5 L copies/ml) were tested by the Abbott m2000rt Real Time and GeneXpert HIV-1 Quant assays. Additionally, 20 seronegative; 16 stored specimens and 10 spiked controls were also tested. Statistical analysis was done using Stata/IC and sensitivity, specificity, PPV, NPV and %misclassification rates were calculated as per DHSs/AISs, WHO, NACO cut-offs for virological failure.

**Results:**

The GeneXpert assay compared well with the Abbott assay with a higher sensitivity (97%), specificity (97-100%) and concordance (91.32%). The correlation between two assays (*r* = 0.886) was statistically significant (*p* < 0.01), the linear regression showed a moderate fit (R^2^ = 0.784) and differences were within limits of agreement. Reproducibility showed an average variation of 4.15 and 3.52% while Lower limit of detection (LLD) and Upper limit of detection (ULD) were 42 and 1,740,000 copies/ml respectively. The misclassification rates for three viral load cut offs were not statistically different (*p* = 0.736). All seronegative samples were negative and viral loads of the stored samples showed a good fit (R^2^ = 0.896 to 0.982).

**Conclusion:**

The viral load results of GeneXpert HIV-1 Quant assay compared well with Abbott HIV-1 m2000 Real Time PCR; suggesting its use as a Point of care assay for viral load estimation in resource limited settings. Its ease of performance and rapidity will aid in timely diagnosis of ART failures, integrated HIV-TB management and will facilitate the UNAIDS 90-90-90 target.

## Background

Success of the ambitious “Treatment 2015” initiative by UNAIDS [1] has accelerated the pace of antiretroviral therapy (ART) scale-up globally. The achievement of this initiative is attributed to the international solidarity and responsibilities shared by diverse disciplines. Upto 2014, an estimated 14.9 million people in developing/low- and middle-income countries received ART [2]. The National AIDS Control Programme in India has successfully reduced the incidence of new HIV-1 infections by 57% and the free roll out of ART has covered nearly 0.8 million HIV infected individuals with nearly 67% coverage of those in need of ART [3]. Furthermore, the wider access to ART has resulted in declining the death rate due to AIDS related co-morbidities [4].

Presently, the free ART programme monitors patients on ART clinically and immunologically with six monthly CD4 count and follows a “Targeted Viral Load” approach for treatment failure. However, several studies have demonstrated poor predictive value of the immunological criteria and reported accumulation of HIV drug resistant mutations due to delayed detection of treatment failure [5–7]. To avoid this, WHO strongly recommends viral load monitoring as the preferred approach to diagnose and confirm treatment failure [8–11].

Nucleic Acid Testing (NAT) based technologies are considered as the gold standards for HIV-1 viral load estimation because of their high specificity, sensitivity and wide linear range of detection. However, these assays require testing to be performed by well-trained technicians and need at least 8-10 h. With an increasing demand to scale up HIV-1 viral load monitoring, a robust, easy to use and sensitive point-of-care technology (POC) [12] is utmost essential. Numbers of POC viral load assays are in the development pipeline [13, 14] and GeneXpert® HIV-1 Quant Assay (Cepheid Innovations Pvt. Ltd., USA) is one of the recently introduced assays. It is a fully automated integrated system that is routinely used for detecting *M. tuberculosis* DNA and resistance to Rifampicin [15, 16] as well as other pathogens (MRSA, Norovirus, HBV, HPV [17–21]) including HIV. It is a rapid assay that delivers results within 90 mins. It uses reverse transcriptase polymerase chain reaction (RT-PCR) technology to achieve high sensitivity over the range of 40 to 10,000,000 copies/ml (1.6 to 7 log_10_copies/ml) and can quantitate all HIV-1 Group M, N and O subtypes [22]. Furthermore, a reduced turnaround time leads to prompt HIV diagnosis and clinical management in adult and pediatric samples. Apart from this, the assay is very simple and safe to perform; their integrated reagents and consumables are robust in usage and can be stored in refrigerators. Due to minimal requirement of infrastructure, it is very easy to scale up the activity of viral load testing nationally. Considering all these features, the Gene Xpert HIV-1 Quant assay serves as a valuable point of care viral load assay that could be useful for monitoring antiretroviral therapy as well as integrated HIV-TB management in developing countries like India [23]. Therefore in the present study, we compared performance of GeneXpert® HIV-1 Quant Assay with the gold standard, routinely used Abbott m2000rt RealTime HIV-1 (Abbott Molecular, Germany) in the resource limited Indian settings in view of the scale up of viral load testing [24]. The performance evaluation of this new assay will ensure its decentralized utility in the clinical set up and will assist in management of patient care efficiently.

## Methods

### Study design

A systematic validation of the GeneXpert® HIV-1 Quant assay (a point-of-care technology) in view of scaling up HIV-1 viral load in India to monitor the success of national ART programme was carried out.

### Ethics statement

The study was approved by the 59^th^ National AIDS Research Institute (NARI) Ethics Committee (Approval No: NARI/EC Protocol No. 2015-10). All study participants provided a written informed consent for collecting demographic information, plasma viral load testing and retention of plasma samples.

### Study setting and specimen collection

The study was conducted from June to September 2015 in HIV-1 positive adult individuals. 314 HIV-1 seropositive (ART naïve *n* = 151, On ART *n* = 129, suspected ART failure *n* = 34; individuals were screened to obtain varying viral load ranges) and 20 normal healthy HIV seronegative individuals were enrolled at three ART centers located in Pune (ART centres: Model Colony; YCM Hospital; Sassoon General Hospital).The whole blood specimens were collected in 10 ml EDTA vacutainers (Becton Dickinson, USA), transported to NARI, centrifuged at 405 g for 10 mins, plasma was separated within 6 h, aliquoted and stored at −70 °C until tested.

### Viral load estimation

Following manufacturers’ instructions, all plasma specimens were tested by an automated Abbott m2000rt RealTime HIV-1 assay (Abbott Molecular Inc., Germany) within 2 days of collection in batches of 24 and results were expressed as copies/ml [25–27].

Of 314, 219 specimens falling in nine viral load ranges (Table 1) were selected and tested by the HIV-1 Quant assay following the manufacturer’s instructions within 3 days after sample collection. Briefly, the assay was carried out by adding 1.2 mL of plasma to the cartridges which contain reagents for RNA extraction, reverse transcription and real-time cDNA quantification based on the conserved region of 3′-LTR of HIV-1.Additionally, following sets 1) spiked copy controls (prepared by spiking the normal human plasma (NHP) with pooled high HIV-1 viral load plasma [*n* = 25, 20 - 20, 00,000 copies/ml] and HIV-1 viral culture supernatant (*n* = 25, 40 - 40, 00,000 copies/ml) and 2) samples stored at three time points (1, 2 and 3 months) were used.

**Table 1:** Viral load ranges selected for validation

Viral Load ranges	Specimens tested by Abbott Real Time HIV-1	Specimens tested by GeneXpert HIV-1 Quant
Not detected	85	27
< 40	20	24
40 -200	24	15
201 -2500	24	16
2501 -5000	21	16
5001 -10,000	23	19
10,001 -1 L	59	59
100,001 -5 L	45	26
> 5 L	13	17
Total	314	219

### Statistical analysis

The viral load values (copies/mL) were transformed to log_10_ copies/mL and the statistical analysis was performed using Stata/IC 10.1 for Windows (Copyright © 1984-2009). The percent concordance among the nine viral load ranges was determined and reproducibility, inter- and intra-assay variation were studied using coefficient of variation. The Bland-Altman plot was generated to assess the limits of agreement and mean bias (95% CI). The descriptive statistical tests were used to compare the misclassifications rates for different viral load cut-offs as per two internationals viz. [Demographic Health Surveys/AIDS Indicator Surveys (DHSs)/AISs) cut-off 200 copies/ml [28] and WHO cut-off 400 copies/ml] [29] and one national cut-off [NACO cut-off 1000 copies/ml] [30] used for classifying the treatment failures. The coefficient of determination (R^2^) was used to assess the linear fit for paired readings of log viral load copies by two methods. Additionally, the lower and upper limits of detection (LLD and ULD) were calculated using minimum and maximum values. The uncertainty limits for natural variation in GeneXpert viral load results were also calculated.

## Results

The paired plasma specimens collected from 219 study participants (mean age 37.6 years, SD = 9.4) were tested by Abbott m2000 Real Time PCR and GeneXpert HIV-1 Quant assays.

### Concordance between Abbott and HIV-1 quant assays

**Fig. 1:**
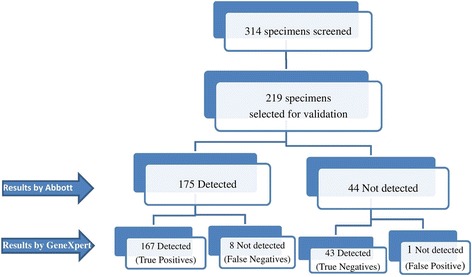
Flow Chart of specimens selected for the validation of GeneXpert HIV-1 Quant

The percentage of concordant samples in both the assays is depicted in Fig. 1 and Table 2. Out of 175 samples detected by the Abbott assay, 8 samples (range: 42-92 copies/mL) were undetectable (false undetected) by the GeneXpert assay. Also, one of the 44 samples undetectable by the Abbott assay, was detectable (42 copies/mL, false detected) in the GeneXpert assay.

**Table 2:**
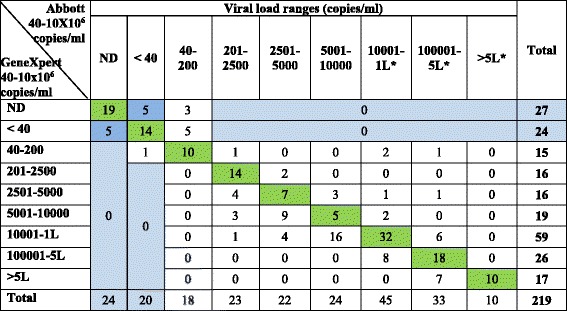
Concordance in different viral load categories of Abbott Real Time HIV-1 and GeneXpert HIV-1 Quant

* Lakhs

The concordance was determined by measuring the percentage of samples containing <40 or ≥40 copies/ml which was 91.32% (200/219 samples). Table 2 shows detailed analysis of the concordance obtained between the two assays. The percent concordance between each of the seven quantifiable categories (40- > 5 L copies/mL) ranged from 82-100%. Out of 167 samples quantifiable by the GeneXpert assay, 86.8% (145/167) of the samples were fitting within the acceptable limit (+/− 0.5 log copies/mL) whereas 13.2% (22/167) were outside this limit. Further analysis of 167 quantifiable samples for studying the correlation between two assays indicated a statistically significant (*p* < 0.01) Pearson’s correlation coefficient (*r* = 0.886).

### Correlation and bland-Altman analysis

**Fig. 2:**
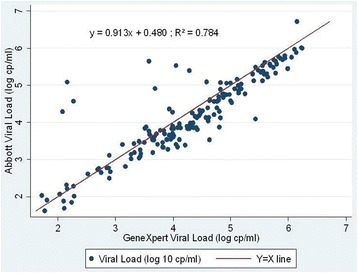
Scatter plot of plasma viral load of Abbott Real Time HIV-1 and GeneXpert HIV-1 Quant

**Fig. 3:**
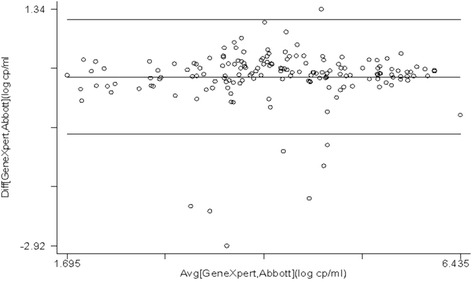
Bland-Altman plot for Abbott Real Time HIV-1 and GeneXpert HIV-1 Quant assay results

The scatter plot indicated a moderate fit with coefficient of determination: R^2^ = 0.784 (Fig. 2). Further, the level of agreement studied using the Bland-Altman plots indicated that the differences were within the limits of agreement (−0.909 to 1.15). No systematic trend was observed from lower to higher values. The mean bias was positive showing an overestimation by the HIV-1 Quant assay with an average bias of 0.120 and 95% CI (0.042 to 0.199) (*n* = 167) (Fig. 3). Overall, results indicated a strong agreement between results of the two assays.

### Sensitivity, specificity, PPV, NPV and percent misclassification

The misclassification data refers to reporting a “Not detected” viral load result as “detected” and vice versa. Considering the implementation of the validated assay on a larger scale, the data was analyzed in view of three cut-offs, DHSs/AIS; WHO and NACO that consider 200, 400 and 1000 copies/ml respectively, which indicated that the sensitivity remained constant (97%) for the three cutoffs while the specificity differed (100, 97 and 98% respectively). The PPV was 100% for the DHS cut off; 99% for WHO and NACO while the NPV showed an increasing trend (88, 89, 91% respectively) (Table 3). The lowest misclassification rate (2.14%) was observed for the cut-off 200 with 95% CI (0.6%, 5.4%). Overall, the percent misclassification rates for the three cut offs were not statistically different when compared by the proportion test (*p* = 0.736).

**Table 3:** Data on sensitivity, specificity, PPV, NPV & % misclassification as per national and international cut-offs

Cut-offs classifying treatment failures	sensitivity, specificity, PPV, NPV & % misclassification
DHSs and AISs cut-off (>200 copies/ml)	Sensitivity = 97%, specificity = 100%, PPV = 100%, NPV = 88% % misclassification (95%CI) = 2.14% (0.6%, 5.4%)
WHO cut-off (>400 copies/ml)	Sensitivity = 97%, specificity = 97%, PPV = 99%, NPV = 89% % misclassification (95%CI) = 2.67% (0.9%, 6.1%)
NACO cut-off (>1000 copies/ml)	Sensitivity = 97%, specificity = 98%, PPV = 99%, NPV = 91% % misclassification (95%CI) = 2.67% (0.9%, 6.1%)

### ULD, LLD, reproducibility analysis and uncertainty limits for the natural variation

The viral load results for 10 paired specimens (with 3 repeat tests) were selected from inter-assay and reproducibility analyses was carried out. The Bland-Altman analysis showed mean difference (max among all) of 0.064 log_10_ copies/ml (95% CI: -0.170, 0.042); which is much lesser than the acceptable limit of 0.5 log_10_ copies/ml. The LLD and ULD for GeneXpert system were 1.62 log_10_ copies/ml. (42 copies/ml) and 6.24 log_10_ copies/ml (1,740,000 copies/ml) respectively.

### Precision analysis on spiked copy controls

The inter- and intra-assay variation assessed using copy controls spiked with pooled HIV-1 viral load plasma indicated variation in the GeneXpert assay as 1.42% (range 0.36–2.74%) and 1.24% (range 0.31–3.34%) respectively across the range of viral load copies (20–20 L copies/ml) (Table 4).

**Table 4:** Inter and Intra Assay Variation between Abbott Real Time HIV-1 and GeneXpert HIV-1 Quant

NHP spiked with high VL plasma					NHP spiked with viral stock				
HIV-1 viral load (copies/ml)	Coefficient of variation (% CV)				HIV-1 viral load (copies/ml)	Coefficient of variation (% CV)			
	Inter-Assay		Intra-Assay			Inter-Assay		Intra-Assay	
	Abbott	GeneXpert	Abbott	GeneXpert		Abbott	GeneXpert	Abbott	GeneXpert
200	4.11	1.97	4.77	3.34	40	1.53	6.96	4.28	2.33
2000	1.08	1.45	1.42	0.31	4000	1.03	3.17	0.77	10.66
20,000	0.86	2.74	2.51	0.97	40,000	0.28	6.84	0.42	0.96
2,00,000	0.57	0.36	1.74	1.11	4,00,000	0.60	3.00	0.49	2.89
20,00,000	0.81	0.58	0.78	0.45	40,00,000	0.69	0.78	0.33	0.78

Additionally, inter- and intra-assay variation assessed using copy controls spiked with HIV-1 viral culture supernatant indicated variation in the GeneXpert assay as 4.15% (range 0.78–6.96%) and 3.52% (range 0.78–10.66%) respectively across the range of viral load copies (40 – 40 L copies/ml) (Table 4).

### Carryover analysis

The possibility of carry-over contamination was verified by placing the 20 seronegative sample before or after the seropositive samples. The results indicated that all seronegative samples were negative without any carry over, indicating 100% specificity of the GeneXpert assay. (Data not shown).

### Linear regression analysis of viral load on stored plasma samples

**Fig. 4:**
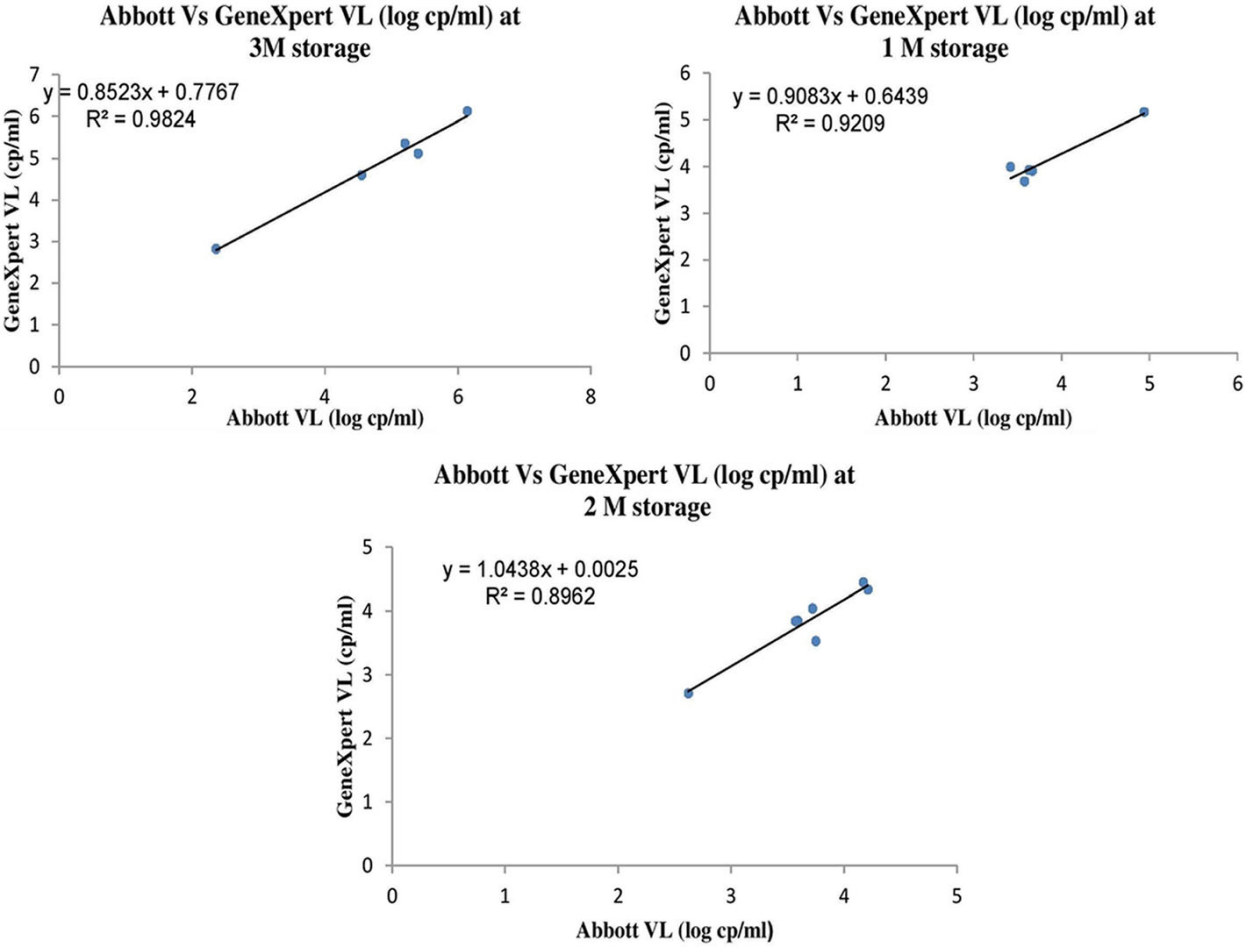
Regression plots for viral load of stored samples for Abbott Real Time HIV-1 and GeneXpert HIV-1 Quant

The linear regression analysis of the samples stored for 1, 2 and 3 months showed a good fit with no significant difference in the regression coefficient [R^2^ = 0.920; 0.896 and 0.982 respectively] (Fig. 4).

## Discussion

NAT based real-time quantitative PCR’s are the preferred options for high-throughput HIV-1 viral-load testing in developed and developing countries [31]. However, these assays are time consuming and flow separately through three steps - sample preparation, amplification, detection and require specimen transportation, high-end instrumentation, skilled personnel, and dedicated laboratories [22]. Hence as suggested by the recent WHO guidelines [11] and to generate results with “sample-in/answer-out” approach [32], replacing NAT based assays with partially/fully automated POC assays is crucial.

The National ART programme in India was launched in April 2004 in limited number of hospitals. Subsequently, concentrated efforts resulted in expansion of the programme. Currently, the programme has adopted a public health approach for provision of ART & provides comprehensive prevention, care and treatment services, with a standardized and simplified combination of ART regimen free of cost to more than 8.8 lakhs PLHIVs across 528 ART centers and 1080 Link ART centers across the country which is a second highest number in the world. Wider access to ART has led to 29% reduction in estimated annual AIDS related deaths [33].

However, the success of high-quality ART depends on timely monitoring of viral load and confirmation of ART failure. Introduction of a simple and reliable POC viral load assay will deliver ART service more appropriately and significantly in a decentralized manner. This will potentially reduce the cost of transportation of samples and monitoring patients on antiretroviral therapy without compromising the quality of care required for treatment success. The “ASSURED” criteria set by WHO describes the POC assays as affordable, sensitive, specific, user-friendly, robust/rapid, equipment-free, and deliverable techniques. Many such POC technologies [13] are in development and GeneXpert HIV-1 Quant assay is one of them. The system requires single-use disposable GeneXpert cartridge that are self-contained and could be useful for viral load monitoring in resource limited settings. As the patient load in Indian ART centers is low and variable, the viral load testing requirement can be low every day in smaller in district ART centers. Such small number of viral load tests are manageable with POC GeneXpert platform [33] In certain districts because the RNTCP, some GeneXpert POC machines for TB diagnosis have already been placed [34] The load of TB testing along with viral load estimation on the same instrument is possible. Hence, convergence of both the program’s is possible through integrated testing.

Our results indicated that HIV-1 Quant assay compared well with the Abbott plasma viral load assay [27, 32]. The high percentages of concordance (91.32%), correlation (*r =* 0.886) and minimum differences in the mean viral loads (0.12 log copies/ml, as measured by Bland-Altman analysis) were observed between the two assays. In comparison to the Abbott assay, the results obtained by the GeneXpert assay for low viral load categories showed an agreement of 79% (19/24) for not detected category, 70%(14/20) for <40 copies/ml category and 100% for 40-200 copies/ml considering number of concordant samples within acceptable limit of 0.5 log copies/ml. Further comparison of the GeneXpert assay showed an agreement of (91, 86 and 83%) in the moderate viral load categories (201-2500, 2501-5000 and 5001-10,000 copies/ml). Whereas in the higher viral load categories (10001-1 L, 100,001-5 L and >5 L copies/ml), the agreement was (87, 82 and 90%). In contrast to the previous studies [35, 36], higher agreements in the viral load categories ranging from 40 to >5 L copies/ml were reported in our studies. Furthermore, it is noteworthy to mention that the LLD (42 copies/ml) obtained in our assays coincided well with the LLD of manufacturer (40 copies/ml).

The main strength of this study lies in the analysis of percent misclassifications based on the international and national cut offs used for classifying the treatment failures. The GeneXpert assay was found to be highly sensitive (91 to 95%) and specific (99 to 100%) and the misclassification rates were not statistically different as per three cut-offs (*p* = 0.736). Also, the study indicated that the kit worked well after 3 months of storage at −70 °C and suggested its robustness in case of the programme implementation while facing hurdles with kit supply, equipment down time, manpower, shortages, etc.

Assay precision and reproducibility were investigated by spiking the seronegative plasma with high viral load plasma/culture supernatant. Our data indicated that the GeneXpert assay showed excellent intra-assay and inter-assay reproducibility at high viral loads (2000-20,000,00 copies/ml), with %CV ranging between 0.31-1.11, 0.36-1.45 except for inter-assay variation for 20,000 copies/ml (%CV 2.74). However, higher variations were observed at low viral loads, ie; at 200 copies/ml (%CV 3.34, 1.97) respectively which was found to be less than the Abbott assay. Thus, variation observed at low viral load results agrees with previously reported study by Swenson et al., in 2014 where comparisons were based on 4221 paired samples [37], These variations could be attributed to the discrepancies in different testing platforms in quantifying subtype C samples as reported earlier [38]. Apart from variations observed while using such platforms, the parameters like type of sample and its integrity were assessed in one of the study wherein good precision as well as accuracy was reported for another POC (Liat HIV Quant (IQuum) platform) in comparison with other platforms (Roche CAP/CTMv2 and Abbott RealTime HIV-1) [39]. In our study, sero-negative samples were included to assess false positives and the carry over effect, which was found to be 100% specific in classifying negative samples as negative with no carryover effect, even if a negative sample preceded a positive specimen.

The main advantages of the GeneXpert HIV-1 viral load assay lies in its simplicity (ease of performance due to its integrated cartridge based closed system), rapid turnaround time (90 min as compared to other viral load platform that require 8-12 h), compactness (equipment is smaller than other platforms), cost effectiveness (17 USD which is cheaper as compared to other assays) and the large portfolio of different assays to manage patients with different variants of HIV-1, TB, and HIV-TB co-infection in the longer run [13, 15]. As nearly 1.1 lakh PLHIVs are reported to have HIV-TB co-infection. India shares one fourth of the global TB burden and one person dies of TB, every 2 min. Keeping in mind, the morbidity due to HIV-TB co-infections, the Indian government has launched the Revised National TB Control Program (RNTCP) in collaboration with NACO (National AIDS Control Organization) and CTD (Central TB Division) that uses WHO recommended Directly Observed Treatment Short Course (DOTS) strategy that is operational in 632 districts/reporting units. It provides daily anti-TB treatment (ATT) for PLHIV at all ART centers, as a single window approach [23, 33, 34]. Prioritization of rapid molecular Cartridge Based GeneXpert assay for all PLHIV with presumptive TB to ensure early diagnosis of TB will help on convergence of load per day for TB where less number of machines is available at district level. Furthermore, both the activities can be best utilized if two programs converge. However, the decision to use one assay or the other could depend on assay-independent parameters such as laboratory space, number of samples and workflow. As per the study design, the predefined viral load ranges quantified using the gold standard Abbott assay obviate the risk of any potential bias for sample selection as it covers varied viral load ranges from low to high viral copies. Furthermore, the inherent capacity of the system for processing 4 samples at a time could serve as a limitation to provide viral load testing for patient care at ART centers that have higher number of patient visits.

Our results suggest that the GeneXpert cartridge based test as an alternative method for viral load monitoring in resource-limited settings, specifically from the rural and remote parts of the country. Our study ascertains the validity and performance of the GeneXpert HIV-1 Quant assay as a point of care technology which could be helpful as reference to other scientists for validating similar POC instruments. Thus, our study results can be an important tool for clinicians, patients, policy makers etc. to scale up the ambitious UNAIDS 90-90-90 treatment target with timely diagnosis of ART failures as well as for management of HIV-TB patients [40]. Further studies of operational issues to apply these findings within a clinical setting on a large scale will be useful in translating into policy decisions that will benefit India and the global community.

## Conclusion

The viral load results of GeneXpert HIV-1 Quant and Abbott HIV-1 m2000 Real Time PCR were comparable. The differences between the HIV-1 viral load estimates for both the assays were within the limits of agreement. Thus, the GeneXpert HIV-1 Quant can be used as a Point of care assay for viral load estimation in resource limited settings. The outcomes from this study appraise the national and international programs for adoption and placement of such integrated platforms for PLHIV and HIV-TB patient care.

## Abbreviations

AIDS: Acquired immunodeficiency syndrome
AISs: AIDS indicator surveys
ART: Antiretroviral therapy
ATT: Antitubercular treatment
CAP-CTM: COBAS® AmpliPrep/COBASO TaqMan®
CI: Confidence interval
CTD: Central TB division
CV: Coefficient of variation
DHSs: Demographic and health surveys
EC: Ethics committee
HBV: Hepatitis B virus
HIV: Human immunodeficiency virus
HPV: Human papilloma virus
LLD: Lower limit of detection
MRSA: Methicillin-resistant *Staphylococcus aureus*
NACO: National AIDS Control Organization
NAT: Nucleic acid amplification technique
NPV: Negative predictive value
PCR: Polymerase chain reaction
PLHIV: People living with HIV/AIDS
POC: Point of care
PPV: Positive predictive value
RNTCP: Revised national TB control program
SD: Standard deviation
TB: Tuberculosis
ULD: Upper limit of detection
UNAIDS: United Nations programme on HIV/AIDS
WHO: World Health Organization
